# A Virus Genetic System to Analyze the Fusogenicity of Human Cytomegalovirus Glycoprotein B Variants

**DOI:** 10.3390/v15040979

**Published:** 2023-04-16

**Authors:** Xuan Zhou, Giorgia Cimato, Yihua Zhou, Giada Frascaroli, Wolfram Brune

**Affiliations:** 1 Leibniz Institute of Virology (LIV), 20251 Hamburg, Germany; 2 Departments of Laboratory Medicine and Infectious Diseases, Nanjing Drum Tower Hospital and Jiangsu Key Laboratory for Molecular Medicine, Medical School of Nanjing University, Nanjing 210008, China

**Keywords:** human herpesvirus 5, glycoprotein B, UL55, cell–cell fusion, entry, infectivity

## Abstract

Viruses can induce the fusion of infected and neighboring cells, leading to the formation of syncytia. Cell–cell fusion is mediated by viral fusion proteins on the plasma membrane of infected cells that interact with cellular receptors on neighboring cells. Viruses use this mechanism to spread rapidly to adjacent cells or escape host immunity. For some viruses, syncytium formation is a hallmark of infection and a known pathogenicity factor. For others, the role of syncytium formation in viral dissemination and pathogenicity remains poorly understood. Human cytomegalovirus (HCMV) is an important cause of morbidity and mortality in transplant patients and the leading cause of congenital infections. Clinical HCMV isolates have broad cell tropism but differ in their ability to induce cell–cell fusions, and little is known about the molecular determinants. We developed a system to analyze HCMV glycoprotein B (gB) variants in a defined genetic background. HCMV strains TB40/E and TR were used as vectors to compare the fusogenicity of six gB variants from congenitally infected fetuses with those from three laboratory strains. Five of them conferred the ability to induce the fusion of MRC-5 human embryonic lung fibroblasts to one or both backbone strains, as determined by a split GFP–luciferase reporter system. The same gB variants were not sufficient to induce syncytia in infected ARPE-19 epithelial cells, suggesting that additional factors are involved. The system described here allows a systematic comparison of the fusogenicity of viral envelope glycoproteins and may help to clarify whether fusion-promoting variants are associated with increased pathogenicity.

## 1. Introduction

All enveloped viruses express proteins needed to fuse the viral envelope with the target cell membrane. This happens either at the cell surface or within an endosome after the internalization of the virion by endocytosis. When the viral proteins that mediate fusion are present on the plasma membrane of an infected cell, the plasma membranes of adjacent cells can merge, leading to the formation of a syncytium. The ability of a virus to induce syncytium formation depends on several factors, such as the quantity of the viral fusion proteins present on the cell surface, the prevalence and accessibility of cellular receptors and coreceptors, and the fusogenicity of the viral fusion machinery [1].

The formation of syncytia can be advantageous or detrimental to a virus. An advantage of cell–cell fusion is that it enables the virus to spread in the absence of extracellular viral particles and thus without the exposure of virions to neutralizing antibodies. However, a disadvantage of cell–cell fusion is the formation of polyploid cells, aberrant mitosis, and cell death due to mitotic catastrophe. These consequences can be detrimental for viruses that replicate slowly or persist for a prolonged period in certain cells. For some viruses, syncytium formation has become an integral part of their replication strategy. Examples are measles virus and respiratory syncytial virus, two members of the *Paramyxoviridae* [2]. Among the *Herpesviridae*, the formation of syncytia is a common observation in varicella-zoster virus (VZV) infection of the skin [3] but is detected only occasionally in cells infected with herpes simplex virus (HSV) or Epstein–Barr virus (EBV). With these viruses, syncytium formation depends largely on strain-specific variants of viral envelope glycoproteins [1,4].

Human cytomegalovirus (HCMV, human herpesvirus 5) is an opportunistic pathogen that causes severe infections in immunocompromised individuals such as transplant recipients [5]. HCMV can be transmitted vertically from mother to child during pregnancy. Congenital HCMV infection is the most common congenital infection worldwide, affecting approximately 1% of all newborns. Up to 15% of congenitally infected children suffer from long-term neurological sequelae such as sensorineural hearing loss or mental retardation [6,7]. Little is known about the viral and host factors determining the likelihood of transplacental virus transmission, the extent of viral dissemination in the fetus, and pathogenicity in the offspring.

The large double-stranded DNA genome of HCMV comprises 230 kbp and encodes more than 200 proteins. Some of the viral proteins show substantial variability [8], suggesting that virus strains with different pathogenicity exist. Viral envelope glycoprotein complexes such as gM-gN, the trimeric and pentameric gH-gL complexes, and gB, which interact with cellular receptors and mediate the fusion of the viral envelope with cellular membranes, have been proposed as determinants of viral genotypes and genotype-associated pathogenicity [9,10]. Results from genome-wide association studies have indeed suggested a correlation between envelope glycoprotein genotypes and viral pathogenicity in congenital infection [11]. However, functional differences of viral glycoproteins in viral attachment, fusion, and entry have not been evaluated as the basis of strain-specific differences in pathogenicity.

HCMV gB, encoded by open reading frame UL55, is the most abundant glycoprotein present on the HCMV envelope [12]. gB is essential for virus entry into target cells and for cell–cell fusion [13,14]. Full-length gB consists of more than 900 amino acids and comprises an N-terminal signal peptide (aa 1–2), an ectodomain with five distinct antigenic domains (domain I to V; aa 23–705), a membrane-proximal region (MPR; aa 706–751), a transmembrane domain (TM; aa 752–796), and a cytoplasmic tail (Cyto; aa 797–906) [14,15]. gB is well conserved across HCMV strains with a mean identity of 96% at the amino acid level [8]. However, different polymorphisms have been described within the N-terminal signal peptide, the disordered domain II loop, and the crown of domain IV [15].

The phenotypic consequences of viral gene variants and mutations can be studied by reverse genetics. The genomes of several HCMV strains have been cloned as bacterial artificial chromosomes (BACs) in *E. coli* and are therefore accessible to genetic engineering by homologous recombination (recombineering) [16]. We have recently identified strain-specific polymorphisms in the HCMV gB responsible for more rapid viral entry into cells and the induction of cell–cell fusion, which may cause instability of the cellular genome [17]. Strain AD169 contains a gB(D275Y) variant, i.e., a tyrosine (Y) residue at position 275 instead of an aspartic acid (D) found in the gB consensus sequence. VR1814, a virus originally isolated from a cervical swab of a pregnant woman and passaged repeatedly on endothelial and epithelial cells [18], contains a gB(S585G) variant. Introducing one of these single amino acid variations into a different HCMV strain was sufficient to confer the syncytium-forming phenotype [17]. Interestingly, a study on clinical HCMV isolates from congenitally infected infants described syncytium-forming phenotypes for some of the isolates [19]. The complete viral genome sequence has recently been published for a few of these isolates [20], and it turned out that three isolates (P4, P14, and P15) contain the same gB(S585G) variant present in VR1814. This finding raised the question of whether syncytium-forming gB variants are highly prevalent in congenital HCMV isolates and whether they might be associated with pathogenicity.

As it is currently not possible to predict the fusogenicity (i.e., the propensity to mediate fusion) of gB solely based on its amino acid sequence, we developed a test system that allows us to analyze different gB variants in a defined genetic background. Here, we describe a convenient gene swap mutagenesis system facilitating the orthotopic insertion of any gB sequence into an HCMV backbone strain by BAC recombineering. We used this system to introduce gB sequences from six fetuses congenitally infected with HCMV and three HCMV reference strains into TB40-BAC4 and TR-BAC. The ability of the recombinant viruses to induce cell–cell fusion in fibroblasts and epithelial cells was quantified using a *Renilla* luciferase–GFP dual split protein (DSP) system. The propensity of the viruses to enter fibroblasts rapidly was analyzed by determining the onset of immediate-early (IE) gene expression, and virus entry by macropinocytosis was analyzed with a specific inhibitor.

## 2. Materials and Methods

### 2.1. Cells and Viruses

MRC-5 human embryo lung fibroblasts (CCL-171), retinal pigmented epithelial ARPE-19 cells (CRL-2302), and human embryonic kidney 293T cells (CRL-3216) were obtained from the American Type Culture Collection (ATCC). MRC-5 and 293T cells were grown in DMEM supplemented with 10% fetal calf serum, 100 U/mL of penicillin, and 100 μg/mL of streptomycin. ARPE-19 cells were grown in DMEM/F-12 GlutaMAX™ (Gibco, Waltham, MA, USA) supplemented with 10% fetal calf serum, 100 U/mL of penicillin, 100 μg/mL of streptomycin, 15 mM of HEPES, and 0.5 mM of sodium pyruvate.

The following HCMV strains were used: VR1814 strain [18,21] and BAC clones of TB40/E and TR [22,23]. Viral stocks were produced from supernatants on infected MRC-5 fibroblasts and concentrated by ultracentrifugation (Beckmann, Indianapolis, IN, USA). First, cells and debris were removed by centrifugation at 5500× *g* for 15 min. Then, the virus was pelleted by centrifugation at 23,000× *g* for 1 h and resuspended in 300 µL of sucrose–phosphate buffer (74.62 g/L of sucrose, 1.218 g/L of K_2_HPO_4_, 0.52 g/L of KH_2_PO_4_) mixed 1:1 with a complete medium. Viral titers were determined on fibroblast cells by using immunofluorescence-based detection of the IE1/2 proteins as described [24].

DNA from the kidneys of congenitally infected fetuses [25] was isolated at the Nanjing Drum Tower Hospital, Nanjing, China.

### 2.2. Plasmids and Reagents

Lentivirus plasmids encoding DSP 1–7 or DSP 8–11 and their helper plasmids pMD-G and pCMVR8.91 have been described [26] and were kindly provided by Dalan Bailey (The Pirbright Institute, Woking, UK). The cloning vector pBR322 was a gift from Adam Grundhoff (Leibniz Institute of Virology, Hamburg, Germany). pEPkan-S and *E. coli* strain GS1783 have been described previously [27]. GS1783 was grown in LB broth (Lennox) containing 5 g/L of NaCl (Sigma-Aldrich, Burlington, VT, USA). Antibiotics were purchased from Roth or Invitrogen and used at the following concentrations: ampicillin (100 μg/mL), kanamycin (50 μg/mL), chloramphenicol (15 μg/mL), and zeocin (25 μg/mL). L-(+)-arabinose was purchased from Sigma-Aldrich. The macropinocytosis inhibitor 5-(N-Ethyl-N-isopropyl)-Amiloride (EIPA) was purchased from Santa Cruz and used at a 50 μM concentration.

### 2.3. Cell–cell Fusion Assay

For the generation of MRC-5 and ARPE-19 cells stably expressing the components of the DSP [28], lentiviruses were produced as described previously [26]. Briefly, HEK 293T cells in a 10 cm dish were transfected by combining 8 μg of plasmid DNA: 3.4 µg of lentiviral vector plasmid, 2.3 μg of pCMVR8.91 (encoding Gag/Pol, Tat, and Rev), 2.3 µg of pMD-G (encoding the vesicular stomatitis virus G protein), and 32 μL of polyethylenimine transfection reagent (Sigma). The growth medium was replaced 8 h post-transfection. At 48 and 72 h post-transfection, supernatants containing lentiviruses were collected, passed through a 0.45 μm microfilter (Roth), and stored at −80 °C. For the transduction of MRC-5 and ARPE-19 cells, lentivirus-containing supernatants were combined with polybrene (Sigma-Aldrich, 5 µg/mL final concentration) and added to cells. After 6 h, the medium was replaced by a complete growth medium, and cells were incubated for another 24 to 48 h. Cells were then expanded and selected with 1 µg/mL of puromycin (Sigma).

For the fusion assay, equal numbers of cells (MRC-5 or ARPE-19) transduced with DSP 1–7 or DSP 8–11 were mixed and seeded, infected with HCMV at an MOI of 1, and incubated for 3 days. To quantify *Renilla* luciferase expression, cells were washed with phosphate-buffered saline (PBS) followed by incubation with coelenterazine-h (Promega) at a final concentration of 2.5 nM. Luminescence was measured on a multi-mode microplate reader (FLUOstar Omega, BMG LABTECH, Ortenberg, Germany). To observe the GFP expression, cells were fixed with methanol/acetone, stained with DAPI to visualize nuclei, and analyzed by confocal microscopy.

### 2.4. Mutagenesis of HCMV Genomes

Full-length UL55 sequences from congenital isolates and reference strains were introduced into the genetic backbones of TB40-BAC4 and TR-BAC by BAC mutagenesis as described in the results. First, the complete UL55 ORF was deleted and replaced by a zeocin (Zeo) resistance marker essentially as described [29], resulting in BACs TB40_ΔgB and TR_ΔgB. Then, full-length UL55 sequences from congenital isolates and reference strains were PCR-cloned in a pBR322-derived low-copy-number plasmid containing the kan/I-SceI cassette from pEPkan-S and a 50 bp homology arm (HA) corresponding to the sequence upstream of UL55.

The fragments containing UL55 with the two HAs and the kan/I-SceI cassette were isolated from the pBR322_kan_gB(X) plasmid by digestion with NotI and BamHI and purified by electrophoretic separation and gel extraction using a NucleoBond Gel and PCR Clean-up kit (Macherey-Nagel, Düren, Germany). Electrocompetent GS1783 bacteria containing the TB40 or TR BAC were electroporated to introduce the purified fragment for the first recombination step. Bacterial clones containing recombinant BACs were grown on LB agar plates with chloramphenicol and kanamycin. In the second recombination step, the kan/I-SceI cassette was removed by arabinose treatment to induce I-SceI expression and cleavage according to the *en passant* mutagenesis protocol [27]. Recombinant TB40/E and TR strains were constructed using BAC mutagenesis as shown in the results. All modified BACs were examined by restriction fragment analysis and sequencing of the modified region. The complete genome sequences of TB40-BAC4 (accession no. EF999921), TR-BAC (accession no. AC146906), and VR1814 (accession no. GU179289) are available at GenBank.

### 2.5. Immunoblot and Immunofluorescence Analysis

Immunoblotting was performed according to standard protocols. At different times post-infection, cells were lysed with RIPA buffer (25 mM of Tris-HCl, pH 7.6; 150 mM of NaCl; 1% NP-40; 1% sodium deoxycholate; 0.1% SDS). Samples were separated by SDS-PAGE and then transferred onto nitrocellulose membranes (Bio-Rad) by semidry blotting. Proteins of interest were detected with specific primary antibodies and secondary antibodies coupled to horseradish peroxidase (DakoCytomation).

Immunofluorescence analysis was performed as previously described. For visualization of syncytia using the DSP system, MRC-5 cells were grown on μ-slides (8-well, Ibidi). After 72 h infection, cells were fixed with methanol/acetone, blocked with 1% milk in PBS, and incubated with specific primary antibodies overnight at 4 °C or for 2 h at 37 °C and secondary antibodies coupled to AlexaFluor 647 (Life Technologies). DAPI (4′,6-diamidino-2-phenylindole, Sigma) was used to stain nuclear DNA. Images were acquired using a Nikon A1+ LSM confocal microscope. For investigating the infectivity, ARPE-19 cells were seeded in 96-well plates. At 72 h post-infection, cells were stained as above. Fluorescence images were acquired using a CellInsight CX5 High-Content Screening Platform (Thermo Fisher Scientific, Waltham, MA, USA) and the percentage of IE-antigen-positive cells was determined by HCS Studio software.

Mouse monoclonal antibodies recognizing HCMV IE1/2 (3H4) and pp150 (XPA 36-14) were generously provided by Thomas Shenk (Princeton University, Princeton, NJ, USA) and William Britt (University of Alabama, Birmingham, AL, USA). A β-actin-specific antibody (AC-74) was purchased from Sigma.

Integrated densities of immunoblot bands were determined for the viral proteins and β-actin using ImageJ (https://imagej.net). The ratios (viral protein vs. β-actin) were determined relative to one reference band (16 hpi without EIPA treatment) for each blot individually and are shown below the blots.

### 2.6. Quantification of HCMV Genomes

HCMV genome copies were determined with a QuantStudio 3 (Thermo Fisher Scientific) qPCR machine essentially as described [17].

## 3. Results

### 3.1. Polymorphism of HCMV Glycoprotein B

**Figure 1:**
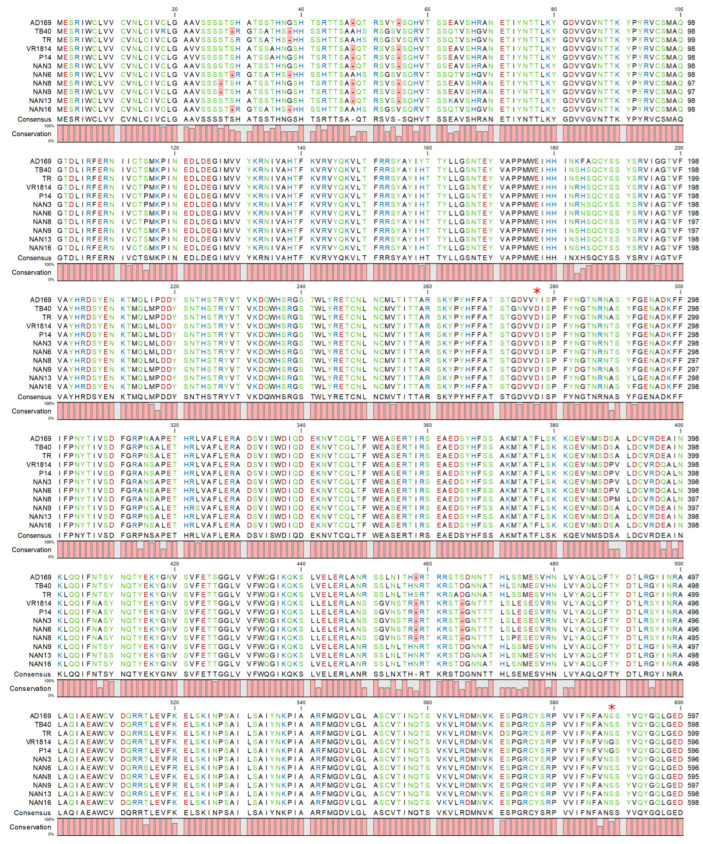

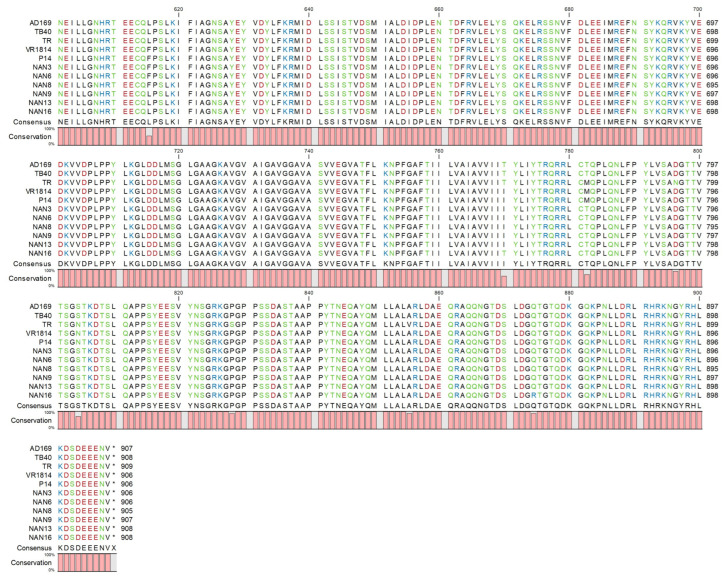
Amino acid sequence alignment of HCMV gBs discussed in this study. The D275Y and S585G variants are marked with an asterisk (*).

HCMV gB is encoded by open reading frame (ORF) UL55. Its amino acid sequence is highly conserved among HCMV strains, but there are frequent polymorphisms in the N-terminal part (containing the signal peptide) and scattered sequence variations throughout the entire protein. We have recently shown that single amino acid variations can be sufficient to change the properties of gB, e.g., resulting in a more fusogenic gB that promotes cell–cell fusion [17]. Interestingly, three congenital HCMV isolates from Italy, described by Galitska et al. [19,20], carried the same gB(S585G) variant that was found to mediate cell–cell fusion in HCMV strain VR1814 [17]. Therefore, we wondered whether highly fusogenic gB variants were frequently present in congenital HCMV isolates. We PCR-amplified and sequenced the HCMV UL55 from DNA extracted from the kidneys of six congenitally infected fetuses for which pregnancy had been terminated because of severe malformations. These cases from the Nanjing Drum Tower Hospital, China, have been described in a previous study [25]. The gB amino acid sequences of the six congenital HCMV strains from Nanjing were aligned to the gB sequences of three Italian congenital isolates and four reference strains: AD169, VR1814, TB40/E, and TR (Figure 1). AD169 and VR1814 carry the previously characterized D275Y and S585G variants, respectively, and the Italian isolates P4, P14, and 15 carry the same S585G variant as VR1814 (Figure 1, only P14 is shown. D275Y and S585G appear at positions 277 and 588, respectively, due to gaps in the N-terminus.). The six gB sequences from the Chinese strains differed in several amino acid positions from the consensus sequence, but none of the sequence variations corresponded to a previously characterized variant. Hence, it was impossible to predict whether these variations have any impact on the biological function of gB.

### 3.2. Development of a Genetic system to Analyze the Functional Differences of gB Variants

We wanted to develop a system allowing the functional comparison of HCMV glycoprotein variants in the context of viral infection. Direct comparison of different HCMV strains in infection experiments provides only limited information because strains differ in many genes and proteins. Therefore, it is very difficult to attribute phenotypic differences to sequence variations in a single gene. Moreover, very few HCMV strains have been cloned as BACs, meaning that the vast majority of strains and isolates are not accessible to modification by site-directed mutagenesis. To address this problem, we decided to use BAC clones of two well-characterized HCMV strains, TB40-BAC4 and TR [22,23], as vector backbones to detect and analyze functional differences of gB.

**Figure 2:**
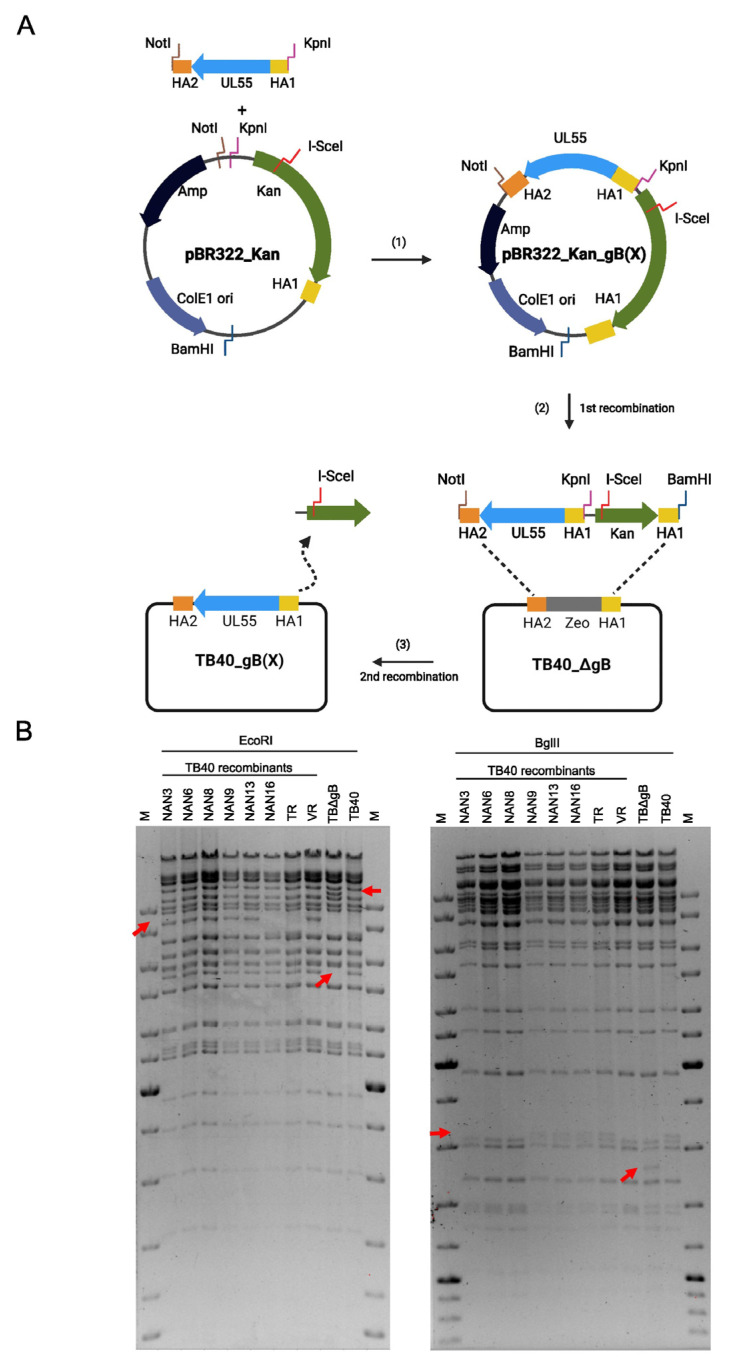
Construction of recombinant HCMV TB40 strains by BAC mutagenesis. (**A**) An optimized workflow was designed for the exchange of gB. (**B**) Restriction fragments of the parental TB40 BAC, TB40_ΔgB, and recombinant BACs carrying the gB of other strains. Expected differences in the restriction patterns are indicated by arrows.

First, a shuttle vector was constructed containing the origin of replication (ori) and the ampicillin resistance (Amp) gene of the low-copy plasmid vector pBR322. This vector was chosen because some viral ORFs, such as UL55, are difficult to clone in high-copy plasmid vectors. The pBR322 fragment was combined with a kanamycin resistance (Kan) marker and an I-SceI endonuclease restriction site from pEPkan-S [27] and a 50 bp homology arm (HA1) identical to the sequence upstream of UL55 (Figure 2A). The full-length UL55 ORF was PCR-amplified from different HCMV strains using primers with 50 bp homology arms up- and downstream of UL55 (HA1 and HA2, respectively). The cloned UL55 ORFs were sequence-verified and then excised from the vector, together with the Kan marker and the homology arms. The linear fragment was used for *en passant* BAC mutagenesis [27] of TB40-BAC4 in *E. coli* strain GS1783. In the first step, the linear fragment was recombined with TB40ΔgB, a TB40-BAC4 carrying a zeocin resistance (Zeo) marker in place of UL55, using kanamycin for positive selection. In the second step, expression of the I-SceI endonuclease was induced with arabinose, resulting in a recombination of the two HA1 sites and excision of the Kan marker. The resulting strain was designated TB40_gB(X), with X referring to the strain from which gB was obtained (Figure 2A).

With this system, eight recombinant TB40 strains were generated that carried the UL55 ORFs of the six congenital HCMV strains from Nanjing and the two reference strains VR1814 and TR (Figure 2B). Analogously, we generated a set of recombinant TR BACs. The infectious virus was reconstituted by the transfection of MRC-5 fibroblasts with the recombinant TB40 and TR BACs, and virus stocks were prepared.

### 3.3. Adaptation of a DSP Reporter System for the Detection of HCMV-Induced Cell Fusion

**Figure 3:**
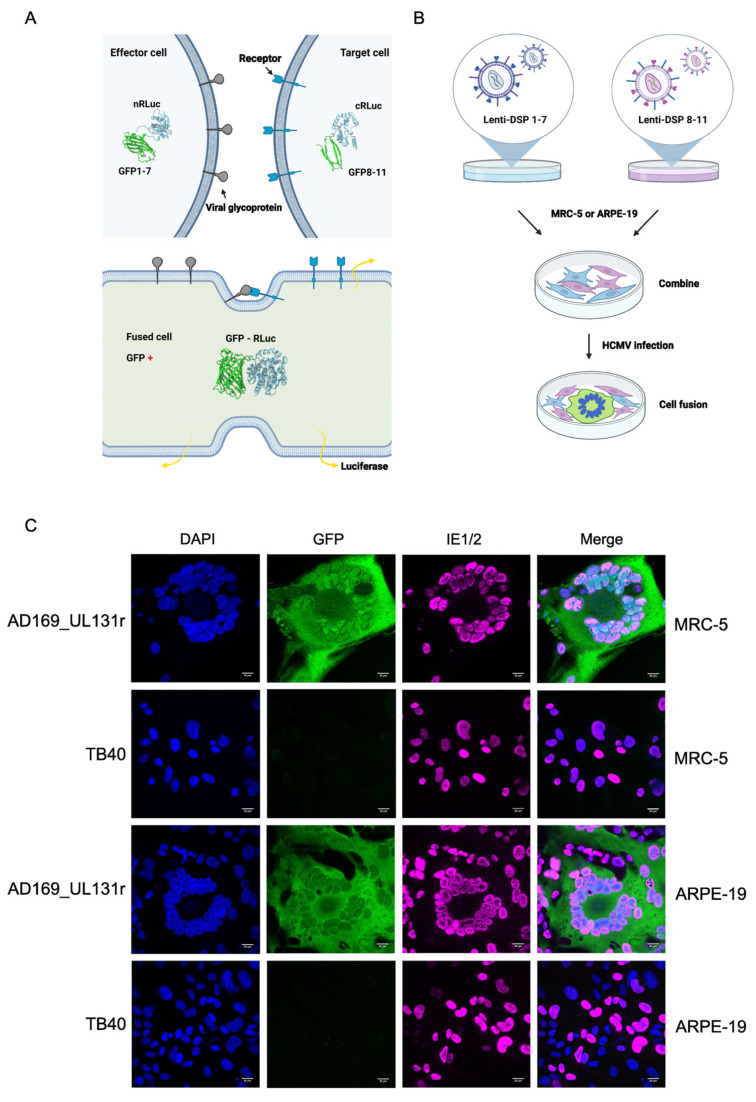
Adaptation of the RLuc-GFP dual split protein (DSP) reporter system to detect HCMV-induced cell–cell fusion. (**A**) Fusion of cells expressing DSP 1–7 and DSP 8–11, respectively, leads to complementation of the split GFP and RLuc proteins. (**B**) Generation of DSP-expressing MRC-5 and ARPE-19 cells by lentiviral transduction. (**C**) Visualization of syncytia using the DSP system. Syncytia containing the complete RLuc-GFP are green fluorescent. The IE1 and IE2 proteins were detected by indirect immunofluorescence, and nuclei were stained with DAPI. Scale bar, 20 µm.

To analyze and compare the fusogenicity of the different gB variants, we wanted to use a reporter system that allows easy visualization and quantification of cell–cell fusion. The *Renilla* luciferase (RLuc)–GFP dual split protein (DSP) system (Figure 3A) appeared to be an attractive system, particularly since it has recently been used to analyze cell–cell fusion induced by respiratory syncytial virus and Nipah virus, two paramyxoviruses [26]. To use this system for studies on HCMV-induced cell–cell fusion, we transduced cells permissive for HCMV infection and replication with lentiviral vectors expressing either one or the other half of the DSP. Transduced cells were selected with puromycin and expanded for use in subsequent experiments. Equal numbers of cells expressing DSP 1–7 and DSP 8–11, respectively, were mixed, seeded in cell culture dishes, and infected with different HCMV strains (Figure 3B). By using strains with known properties, we verified that this system was suitable for the detection of HCMV-induced syncytium formation. Strain AD169 with a repaired UL131 ORF (UL131r), which is known to induce large syncytia [30], induced syncytium formation in MRC-5 fibroblasts and ARPE-19 epithelial cells, whereas strain TB40 did not (Figure 3C).

### 3.4. Analysis of the Fusogenicity of gB Variants from Congenital HCMV Strains

**Figure 4:**
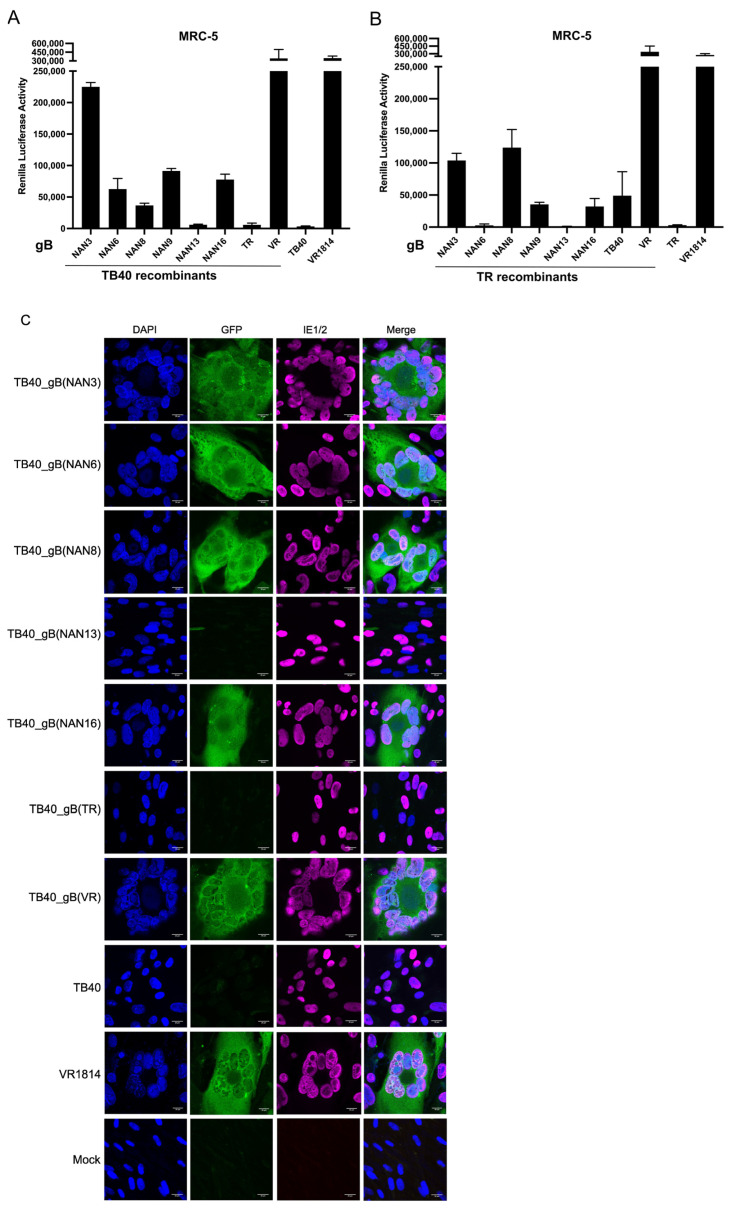
HCMV-induced cell–cell fusion of MRC-5 fibroblasts. Cells expressing the DSP system were infected at an MOI of 1. Cell–cell fusion was detected 3 days post-infection by measuring *Renilla* luciferase activity (**A**,**B**) or by the detection of GFP fluorescence (**C**). The bar diagrams show means ± SD of three biological replicates. Scale bar, 20 µm.

Next, we used the DSP system to determine the fusogenicity of gB variants from congenital HCMV strains. MRC-5 cells transduced with the DSP system were infected with the recombinant TB40 and TR strains described above (Figure 2). Five of the six gBs from the Nanjing congenital strains induced the fusion of MRC-5 fibroblasts when expressed by recombinant TB40 strains, as measured by *Renilla* luciferase activity (Figure 4A). The fusogenicity of gB(NAN3), gB(NAN6), gb(NAN8), gB(NAN9), and gB(NAN16) was higher than the fusogenicity of gB(TB40) and gB(TR) but lower than the fusogenicity of gB(VR). The recombinant TR strains also induced the fusion of MRC-5 cells (Figure 4B), but the extent of fusion differed from the one measured with the TB40-based strains, suggesting that cell–cell fusion depends not only on gB but also on additional strain-specific factors. Cell–cell fusion also resulted in GFP reconstitution in syncytia, as detected by fluorescence microscopy (Figure 4C).

**Figure 5:**
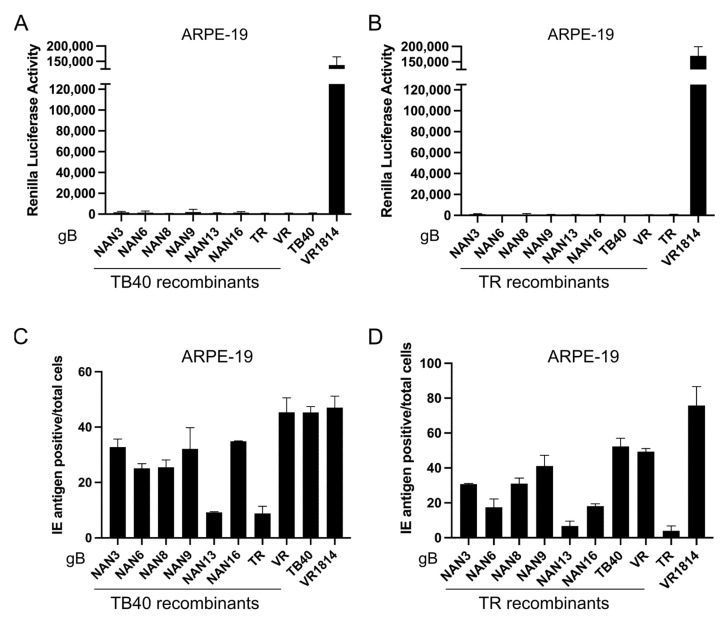
Cell–cell fusion and infectivity in ARPE-19 epithelial cells. (**A**,**B**) ARPE-19 cells expressing the DSP system were infected at an MOI of 1. *Renilla* luciferase activity was measured 3 days post-infection. (**C**,**D**) ARPE-19 cells were infected at an MOI of 1. The percentage of IE-antigen-positive cells was determined 3 days post-infection by immunofluorescence. The bar diagrams show means ± SD of three biological replicates.

To find out whether the recombinant strains could induce syncytium formation in epithelial cells, we infected ARPE-19 cells previously transduced with the DSP system. While strain VR1814, which is known to induce syncytia in this cell type [31], induced very high luciferase activity in infected ARPE-19 cells, none of the recombinant TB40 and TR strains induced luciferase activity indicative of syncytium formation (Figure 5A,B). Interestingly, gB(VR) was not sufficient to induce epithelial cell fusion when expressed by recombinant TB40 or TR strains.

The HCMV strains used in this study had been titered on fibroblasts. Thus, the inability to induce epithelial cell fusion might have been caused by the poor infectivity of the strains on epithelial cells. To rule out this possibility, we determined the infectivity of all strains on ARPE-19 epithelial cells. Cells were infected at an MOI of 1 and the percentage of infected cells was determined 3 days post-infection by staining for IE antigens. As shown in Figure 5C,D, the infectivity varied between strains. Many of the recombinant TB40 and TR strains were similar to VR1814 in terms of infectivity but did not induce cell–cell fusion in ARPE-19 cells (Figure 5A,B). The strains with the lowest infectivity on ARPE-19 cells were those expressing gB(NAN13) and gB(TR). From these results, we concluded that gB variants capable of promoting the fusion of MRC-5 fibroblasts were not sufficient to trigger the fusion of ARPE-19 epithelial cells, suggesting that additional factors besides gB are needed for the fusion of epithelial cells. However, the gB variants had an influence on virus infectivity in ARPE-19 epithelial cells.

### 3.5. Influence of gB Variants on Virus Entry

**Figure 6:**
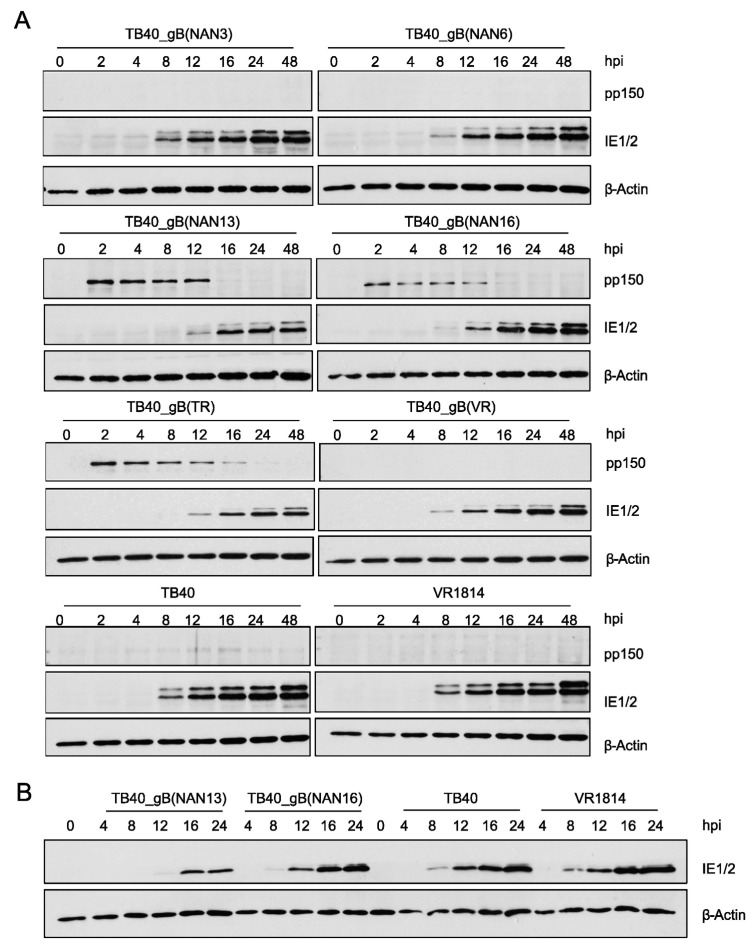
Infectivity of recombinant TB40 strains. (**A**) MRC5 cells were HCMV-infected at an MOI of 0.5. The viral tegument protein pp150 and the IE1 and IE2 proteins were detected by immunoblot analysis. (**B**) For better comparison, selected samples shown in panel A were separated and analyzed on the same polyacrylamide gel. hpi, hours post-infection.

Since gB plays an important role in viral infectivity and entry, we tested whether TB40E and TR mutants show differences in the kinetics of viral IE gene expression. MRC-5 fibroblasts were infected at an MOI of 0.5 with recombinant TB40 and TR strains and control strains. At different times post-infection, the viral tegument protein pp150 and the viral IE1 and IE2 proteins were detected by immunoblot (Figure 6A and Supplementary Figure S1B). We noticed large differences in the amounts of the pp150 tegument protein, which is brought into the cells by the viral inoculum. The strains with the poorest infectivity on ARPE-19 cells (i.e., the ones expressing gB(NAN13) and gB(TR)) also brought in the largest quantities of pp150. We also determined the genome:infectious unit (IU) ratios for the recombinant TB40 strains (Supplementary Figure S1A) by quantitative PCR, and the results supported the conclusions drawn from the pp150 blots. These findings indicated that these viruses also have poor infectivity on fibroblasts as a much larger quantity of viral particles was needed to attain the same titer.

We also observed that expression of IE1 and IE2 proteins was clearly detectable at 8 h post-infection (hpi) in strains with high infectivity (low pp150 levels) but only at 12 to 16 hpi in strains with low infectivity (high pp150 levels) (Figure 6A and Supplementary Figure S1B), suggesting that viral entry and/or the onset of viral IE gene expression was delayed in the strains with low infectivity. To confirm this observation, samples from selected strains were loaded onto the same gel and evaluated by immunoblot analysis. Again, IE protein expression was detected as early as 8 hpi in strains with high infectivity (TB40 and VR1814) but only at 12 hpi (TB40_gB(NAN16)) or 16 hpi (TB40_gB(NAN13)) in strains with intermediate or low infectivity, respectively (Figure 6B and Supplementary Figure S1C).

HCMV can enter fibroblasts by the fusion of the viral envelope with the plasma membrane [32,33] or macropinocytosis [34]. Entry by macropinocytosis can be blocked by treatment with the macropinocytosis inhibitor ethyl-isopropyl amiloride (EIPA) [17,34,35]. To test the influence of EIPA treatment on virus entry, we infected fibroblasts with recombinant TB40 and control strains in the absence or presence of EIPA. IE protein expression was analyzed by immunoblot and used as a measure of the virus’ ability to enter cells and initiate viral gene expression. Relative IE protein levels were quantified by densitometry.

**Figure 7:**
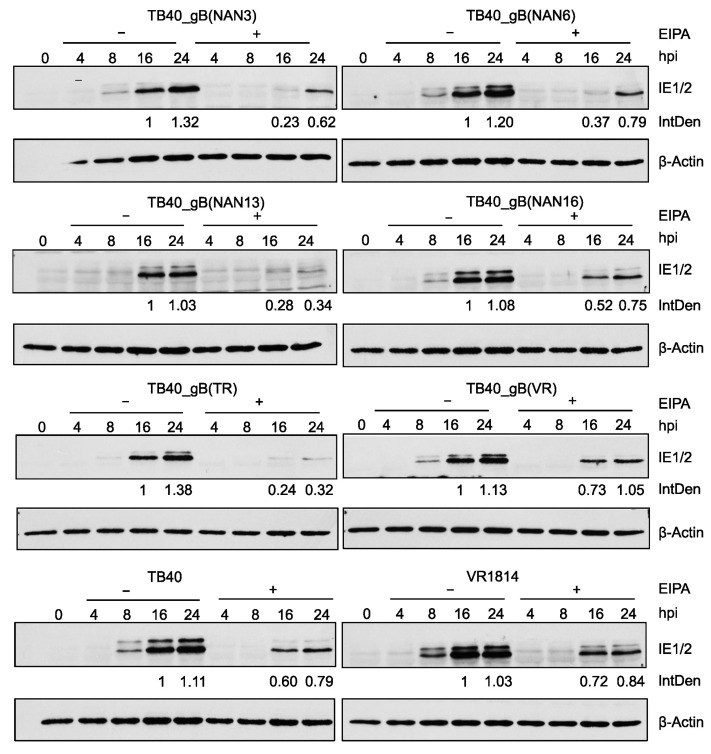
Inhibition of virus entry and IE gene expression by the macropinocytosis inhibitor EIPA. MRC-5 cells were pretreated for 30 min with 50 μM of EIPA and infected in the presence of EIPA at an MOI of 0.5. Two hours post-infection (hpi), EIPA was removed and fresh medium was added. Cells were harvested at the indicated times and IE1/IE2 expression was analyzed by immunoblot. IE1 expression levels at 16 and 24 hpi were measured by densitometry and normalized to β-actin levels. Numbers indicate IE protein levels relative to the levels at 16 hpi in untreated (−EIPA) cells. IntDen, integrated density.

As shown in Figure 7, all tested strains were inhibited to some extent by EIPA, suggesting that entry by macropinocytosis plays an important role. However, the strongest inhibition by EIPA was observed with TB40_gB(NAN13) and TB40_gB(TR), suggesting that these strains are more dependent on macropinocytosis for entry and less capable of utilizing other entry pathways such as envelope fusion with the plasma membrane.

## 4. Discussion

In this study, we developed a straightforward genetic system for the functional analysis of viral gene variants in a defined genetic background. We used this system to analyze gB variants from six congenital HCMV strains and three reference strains in the genetic background of HCMV strains TB40 and TR. The system is simple and fast as it requires only one PCR-cloning step and one round of *en passant* BAC mutagenesis (Figure 2A). This system can easily be adapted for the analysis of other viral glycoprotein genes or other viral genes in general. A similar gene swap approach was recently used to analyze variants of HCMV glycoprotein O (gO) [36].

The recombinant TB40 and TR strains were used to analyze the fusogenicity of the different gB variants, i.e., their ability to induce cell–cell fusion. In MRC-5 human embryonic lung fibroblasts, the gB(VR) vigorously induced cell–cell fusion (Figure 4), regardless of the genetic background in which it was expressed (TB40, TR, or VR1814). The gBs of five Nanjing congenital HCMV strains induced substantial levels of cell–cell fusion in the TB40 background and to a lesser and more variable extent in the TR background (Figure 4). In contrast, gB(NAN13) was not fusogenic in either background nor was gB(TR). Recombinant viruses expressing gB(NAN13) or gB(TR) also had low infectivity on both MRC-5 and ARPE-19 cells, whereas gB(TB) conferred high infectivity (Figure 5 and Figure 6). Overall, infectivity and propensity to induce cell-cell fusion appear to be related but discernable properties of gB, that are influenced by the viral genetic background. A similar dependence on the viral genetic background has been reported for gO variants (genotypes) expressed in HCMV strains TR and Merlin [36].

While infectivity and the ability to induce the cell–cell fusion of MRC-5 cells were largely gB-dependent, the cell–cell fusion of ARPE-19 was not. Strain VR1814 induced syncytia in ARPE-19 cells, consistent with a previous report [31], but the transfer of gB(VR) was not sufficient to confer this property to other strains (Figure 5). It seems likely that other factors are involved, such as the trimeric or the pentameric gH-gL complexes, which cooperate with gB to allow infection of different cell types [37]. However, other less obvious viral genes might also be involved in the regulation of epithelial cell fusion. A recently isolated variant of strain TB40/E, called TB40/EE, induced large syncytia in ARPE-19 cells [38], and a genome sequence comparison revealed several nonsynonymous substitutions in nonglycoprotein genes, compared to the related nonsyncytiogenic TB40/EF variant [39].

HCMV entry into cells is thought to occur either by the fusion of the viral envelope with the plasma membrane or by endocytosis (macropinocytosis) followed by envelope fusion with the endosomal membrane [13,32,33,34,40]. Both routes have been described for HCMV entry into fibroblasts, but their relative contribution and their dependence on gB variants remain largely unknown. A recent study showed that AD169L_gB(275Y) entered fibroblasts more rapidly than AD169_gB(275D). Entry of AD169L_gB(275Y) was barely inhibited by the macropinocytosis inhibitor EIPA, whereas entry of AD169_gB(275D) was strongly inhibited [17], suggesting that the entry of the latter occurred predominantly by macropinocytosis. Of the strains analyzed in this study, the ones carrying gB(NAN13) and gB(TR) were the most sensitive to inhibition by EIPA (Figure 7), suggesting that entry occurred predominantly by macropinocytosis. However, more detailed analyses with different assays and inhibitors will be necessary to come to a definitive conclusion.

Galitska et al. analyzed the biological properties of 21 HCMV isolates from congenital and post-natal infections and found that several isolates formed enlarged flower-shaped syncytial foci in infected cells [19]. However, the genetic basis for this phenotype has remained unclear. More recently, the same authors published the complete genome sequence of five of these HCMV isolates, among them the syncytium-forming isolates P4, P14, and P15 [20]. When we aligned the gB amino acid sequences of these three isolates to known gB sequences, we noticed that all three had a gB identical to that of VR1814, an HCMV known to form large syncytia in MRC-5 and ARPE-19 cells. This prompted us to analyze gB variants from six congenital HCMV strains from Nanjing, China. We found that five of them promoted syncytium formation in MRC-5 cells when expressed in the TB40 background and three in the TR background. The recombinant TB40 and TR strains did not induce syncytia in ARPE-19 epithelial cells, suggesting that the induction of epithelial cell fusion requires additional viral factors. Unfortunately, the Nanjing congenital HCMV strains could not be analyzed directly in terms of infectivity and fusogenicity because only DNA isolated from infected fetal tissue was available. However, the streamlined gene swap system described here should make it feasible to analyze other envelope glycoproteins that are required for the infection of epithelial cells, such as gH, gL, and UL128-131. The important question of whether syncytial phenotypes are highly prevalent or overrepresented in congenital HCMV strains cannot be answered yet as the number of congenital and control strains analyzed thus far is too small. However, the methods described in this study should make broader studies feasible.

## Data Availability

Data are contained within the article or Supplementary Material.

## Supplementary Materials

The following supporting information can be downloaded at: https://www.mdpi.com/article/10.3390/v15040979/s1, Figure S1: Infectivity of recombinant strains.
