# Supplementary material for: A Virus Genetic System to Analyze the Fusogenicity of Human Cytomegalovirus Glycoprotein B Variants
Source: Viruses. 2023 Apr 16;15(4):979. doi: 10.3390/v15040979 (PMC10142178; doi:10.3390/v15040979)
Supplement: Supplementary file 1 [file viruses-15-00979-s001.zip › viruses-2283686-supplementary.pdf]

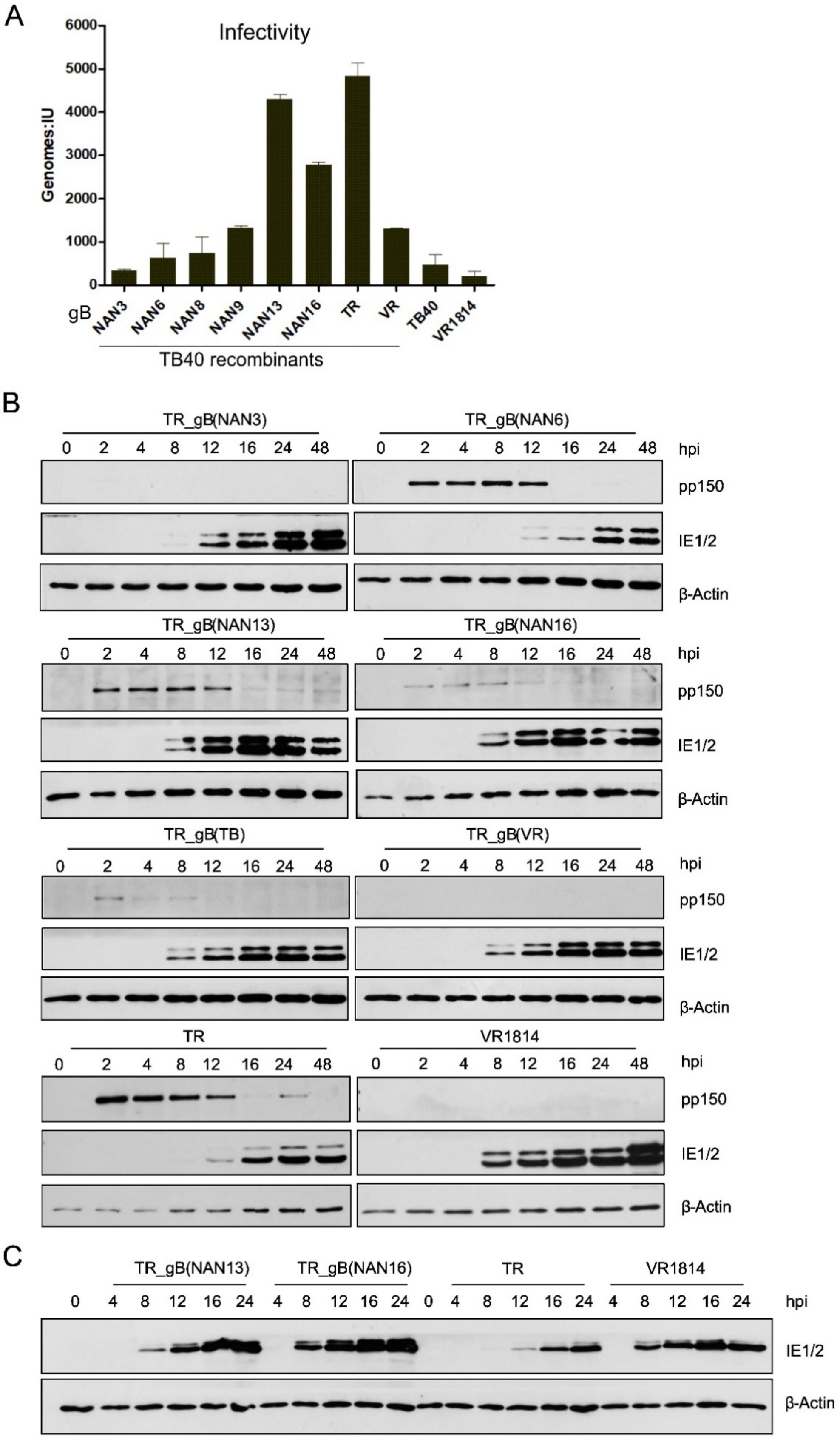

Supplementary Figure S1. Infectivity of recombinant strains. (A) Genomes : IU ratios of recombinants TB40 strains. Means ± SEM of three replicates are shown. (B, C) Infectivity of recombinant TR strains. (B) MRC-5 cells were HCMV-infected at an MOI of 0.5. The viral tegument protein pp150 and the IE1 and IE2 proteins were detected by immunoblot analysis. (C) For better comparison, selected samples shown in panel A were separated and analyzed on the same polyacrylamide gel.
